# 

**Published:** 1888-01

**Authors:** 


### CONTRIBUTORS TO VOL. IX.

E. H. Bradley, V. S.
Richard W. Burke, M. R. C. V. S., A. D. V.
P. Z. Colsson, V. S.
J. T. Duncan, M. D.
Wm. Alston Edgar, F. R. C. V. S.
Robert Formad, D. V. S.
Rush S. Huidekoper, M. D. Vet.
Daniel D. Lee, M. D. V.
John Lindsay, D. V. S.
Charles C. McLean, V. S.
Cornelius M. O'Leary, M. D., L. L. D
W. H. Pendry, D. V. S.
Thomas B. Rogers, D. V. S.
D. E. Salmon, D. V. S.
Robert Meade Smith, M. D.
William Sterling, V. S.
N. E. Brill, A. M., M. D.
L. C. Wakefield, M. R. C. V. S.
A. W. Clement, V. S.
W. A. Conklin, Ph. D.
Henry F. Formad, B. M., M. D.
William S. Gottheil, M. D.
George K. Kinnell, M. R. C. V. S.
Joseph Leidy, M. D., L. L. D.
Henry O. Marcy, A. M., M. D., L. L. D.
T. Wesley Mills, M. A., M. D.
William Osler, M. D.
Leon N. Reefer, V. S.
H. E. Rowell, V. S.
R. W. Shufeldt, M. D.
Edward C. Spitzka, M. D.
G. Archie Stockwell, M. D.
Thomas Walley, M. R. C. V. S.

### INDEX.

Abdominal puncture........ 193
Abscess, a large........ 324
in the brain of a horse........ 193
Academy of veterinary science and comparative pathology.. 204
A common lesion of the horse........ 389
American veterinary college........ 202
review prize........ 95
Army veterinary service, legislation for........ 186
Association for the study of Comparative Psychology...... 423
Ass, wild, parasites in the........ 96

Blood, comparative studies of........ 254
Boa constrictor, some observations on the........ 404
Books and pamphlets received........ 111, 207, 339
Bovine metrotomy........ 191
monstrosity........ 190
Bradley, E. H., death from ovarian tumor........ 409
Brill, N. E., lyssa and the Pasteur fiasco........ 55
British medical association........ 417
Burke, R. W., some rare forms of eye disease in veterinary practice........ 18

Case department........ 188, 322, 405
Castor oil fed lipoma........ 324
Cattle quarantine in Penn........ 187
Chimpanzee, notes on the autopsy of........ 405
Chloral hydras as an anti-spasmodic........ 189
Choera in dog........ 413
Chronic metritis in mare........ 414
Clement, A. W., description of some specimens of pleuro-pneumonia contagosia........ 151
Colon and cæcum, malpositions of........ 113
of the horse, its diseases and derangements........ 1
Colorado state veterinary association........ 202
Colsson, P. Z., death of a cow caused by a piece of wire penetrating heart........ 189
the use of chloral hydras as an anti-spasmodic. 189

Comparative pathology, eczema........ 216
psychology........ 43
some phases of........ 85
studies of mammalian blood........ 254
Congential failure of union of folded and floating colon........ 407
Congress for the study of tuberculoris........ 397
Conklin, W. A., history of the chimpanzee........ 405
Correspondence........ 193, 325
Cow, death caused by wire penetrating heart of........ 189

Death from ovarian tumor........ 409
Descriptions of some specimens of pleuro-pneumonia, contagiosa 151
Diagnosis classification and nature of tumors........ 155
Dog, chorea in........ 413
Domestication and function........ 248
Duncan, J. T., Ontario veterinary college........

Eczema, comparative pathology of........ 216
Edgar, W. A., comparative pathology eczema........ 216
Editorial department........ 85, 177, 315, 307
Emphysema of neck........ 188
Epizootic of glanders in Penn........ 319
Exostosis in cervical vertebræ of horse........ 191
Eye disease, rare forms in veterinary practice........ 18

Formad, H. F., comparative studies of mammalian blood........ 254
the diagnosis classification and nature of tumors 155
Fracture of radius in a bull........ 410
gelding........ 190

Gallus bankiva........ 320
observations upon the morphology of........ 343
Gastric digestion in the hog........ 179
juice of the horse........ 315
Glanders, an obscure case of........ 322
epizootic of........ 319
Gottheil, W. S., autopsy of the chimpanzee........ 406
note on tænia elliptica........ 126
Grouse, physiological relations of gular vocalization in........ 127

Harvard veterinary school........ 171
Histology and pathology of reproduction........ 19
Horse, abscess in the brain of........ 193
a common lesion of the........ 389

Horse, colon of, its diseases and derangements ............... 1
exostosis in cervical vertebræ of ........................ 191
gastric juice of ........................................ 315
œsophagus carried away by sloughing ................ 189
origin of the domestication of ........................ 377
rheumatic affections in the ............................ 323
Hog cholera ................................................ 136
gastric digestion in the .................................. 179
Huidekoper, R. S., account of tumors in domestic animals.... 155
an obscure case of chronic glanders ....... 322
origin of the domestication of the horse... 377
statistics of hospital service ............... 411
Hysteria in mare ............................................ 413

Kinnell, G. N., emphysema of neck ........................ 188
Knuckling in zebra .......................................... 413

Lee, D. D., a common lesion of the horse .................. 389
Harvard veterinary school ......................... 171
Leidy, J., parasites of the shad and herring .................. 211
Lindsay, John, fracture of radius in a bull .................. 410
torsion of the neck of the uterus ..............1 92
Lyssa and the Pasteur fiasco .................................. 55

Malpositions of the colon and cæcum ........................ 113
Marcy, H. O., histology and pathology of reproduction........ 19
Mare, chronic metritis in ........................................ 414
hysteria in ........................................... 413
McLean, C. C., abdominal puncture ............................ 192
bovine metrotomy ............................ 191
monstrosity ........................... 190
compound fracture of radius ................ 408
congenitial failure of union of folded and floating colon ........................... 407
exostosis in cervical vertebræ of horse ........ 191
oblique fracture of radius in gelding .......... 190
rapid union of first phalanx ................... 407
Mills, T. W., comparative psychology ......................... 43
Montreal veterinary college ...................... 78
Montreal veterinary college .................................. 327
and its founder .................. 78

National veterinary sanitary association ..................... 105
New Jersey state veterinary society ..................... 200, 331
New publications ................................................ 320

New York college of veterinary surgeons.................... 203
state academy of veterinary science.............. 204
Notes.................................................... 97
on tænia elliptica.................................. 126
autopsy of the chimpanzee.......................... 126
upon soft-shelled turtles............................ 28

Obituary................................................. 196
Obscure case of chronic glanders......................... 322
Observations on the boa constrictor...................... 404
python............................................ 97
morphology of gallus bankiva............ 343
Oesphagus of horse carried away by sloughing............ 189, 417
Ohio State vetetinary medical association................ 198
O'Leary, C. M., domestication and function................. 248
some phases of comparative psychology...... 85
Ontario veterinary college...................... 107, 204, 328, 392
medical association....................... 202
Origin of the domestication of the horse.................. 377
Osler William, scarlet fever and disease in cattle.............. 177
veterinary department, University of Pa...... 312
Osteology of porzana carolina.............................. 231
Ovarian tumor............................................ 409

Paralysis of bowels treated by electricity.................. 410
Parasites in the wild ass.................................. 96
of the shad and herring............................ 211
Pasieur fiasco and lyssa................................... 55
Peculiar case of rheumatism................................ 323
Physiological relations of gular vocalization in grouse........ 127
Pleuro-pneumonia........................................ 97
contagiosa.................................. 151
Portrait, D. McEachran.................................., 78
Andrew Smith....................................
Proceedings of veterinary societies.............. 103, 197, 327, 415
Psychology, comparative................................ 43
some phases of.................... 85
Python, observations on.................................. 97

Rapid union of fracture of first phalanx..................... 407
Rare forms of eye disease in veterinary practice............. 18
Reever, L. N., failure of union of folded and floating colon... 407
rapid union of fracture of first phalanx....... 407
Reproduction, histology and pathology of..................... 19

Reviews........................................ 108, 205, 332
Rheumatic affections in the horse.......................... 102
Rheumatism, peculiar case of.................................. 323
Rogers, T. B., a peculiar case of rheumatism.................. 323
Rowell, H. E., oesphagus of a horse carried away............ 189

Salicylate of soda in rheumatic affections.................... 102
Salmon, D. E., hog cholera .................................. 136
Selections from foreign journals........................ 100, 428
Scarlet fever and disease in cattle.......................... 177
Shufeldt, R. W., observations upon the morphology of gallus bankiva .................................. 343
oesteology of porzana carolina............... 231
Smith, R. M., gastric digestion in the hog.................... 179
the gastric juice of the horse.................. 315
Snake bite and its antidote.................................. 318
Some observations of the boa constrictor...................... 404
Spitzka, E. C., on brain of the chimpanzee.................... 406
Statistic of hospital service, university of Pa.............. 411
Stirling, William, paralysis of bowels treated by electricity... 410
Stockwell, G. A., notes upon soft-shelled turtles.............. 28
physiological relations of gular vocalization in grouse.................................. 127
Strangulated hæmorrhoids.................................... 414

Tænia. ellipteca, note on.................................... 126
Tetanus original investigation on the ætiology of............. 100
Torsion of the neck of the uterus ............................ 192
Tumors, diagnosis and classification of....................... 155
in domestic animals...................................... 155
Turtles, soft-shelled, notes upon.............................. 28

United States army veterinary service.......................... 95
veterinary medical association.. 94, 103, 186, 197, 415
University of Penna., veterinary department of.......... 312, 327

Veterinary association, Colorado state....................... 202
college, American................................ 202
Montreal................................ 327
and its founder................... 78
Ontario......................... 107, 204, 328
department, University of Penna................ 312
medical association, Ohio state................... 198
Ontario..................... 202
United States.94, 103, 186, 197, 415

Veterinary sanitary association, national.................... 150
school, Harvard.................................... 171
service, U. S. army.................................... 95
society, New Jersey state..................... 200, 331
societies proceedings........................ 103, 415
surgeons, New York college of.................... 203

Wakefield, L. C., castor-oil fed lipoma....................... 324
abscess the result of a punctured wound of the abdomen.................................. 324
Walley, Thomas, malpositions of the colon and cœcum........ 113
the colon of the horse, its diseases and derangements.................................. 1

Zebra, knuckling in.......................................... 413

THE PHYSICIANS' VISITING LIST. P. Blakeston, Son & Co., Philadelphia, 1888.

This handy book is now in its thirty-seventh year of publication and this year comes out with several improvements, the many years experience of the publishers have enabled them to put this together in the best manner.

### BOOKS AND PAMPHLETS RECEIVED

BULLETIN DU MUSEE ROYAL D'HISTOIRE NATURELLE DE BELGIQUE. Tome I., II. and III. Bruxelles, 1882-85.

PROCEEDINGS AND TRANSACTIONS OF THE NATURAL HISTORY SOCIETY OF GLASGOW. Glasgow, 1869-86.

JOURNAL OF THE BOMBAY NATURAL HISTORY SOCIETY. Bombay, 1886-87.

PROCEEDINGS OF THE CANADIAN INSTITUTE. Toronto 1856-87.

JOURNAL OF THE CINCINNATI SOCIETY OF NATURAL HISTORY. VOL. X., No. 3. October, 1887.

PROCEEDINGS NEWPORT NATURAL HISTORY SOCIETY. Newport, 1886-87.

THE PROCEEDINGS OF THE LINNEAN SOCIETY OF NEW SOUTH WALES. VOL. II., PART 2. Sydney, 1887.

ANNALS ET BULLETIN DE LA SOCIETE DE MEDICINE DE GAND. Ghent, 1887.

ORNIS INTERNATIONAL ZEITSCHRIFT, FUR DIE GESAMMTE ORNITHOLOGIE 3rd JAHRGANG HEFT II. and III. Wein 1887.

THE ARCHIVES OF VETERINARY SCIENCE. October, St. Petersburg, 1887.

LE NATURALISTE CANADIEN. REDACTUER M. L'ABBE PROVANCHER. Cap Rouge, 1887.

JOURNAL DE PHARMACIA E SCIENCIAS ACCESSORIAS DE LISBON. Published by Jose Tedesch. Lisbon, 1887.

VEEARTSENIJKUNDIGE BLADEN VAN VEEARTSENIJKUNDE IN NEDERLANDISCH-INDIE. Deel II. aff. 2 Batavia, 1887.

THE MICROSCOPE, AN ILLUSTRATED MONTHLY JOURNAL DEVOTED TO MICROSCOPICAL SCIENCE. Published by D. O. Haynes & Co. Detroit.

BULLETIN ILLINOIS STATE LABRATORY OF NATURAL HISTORY. Part of Vols. II. and III. Champaign.

JOHNS HOPKINS UNIVERSITY CIRCULARS, Nos. 60 and 61. Baltimore.

On a Collection of Birds Sterna and Skulls, by Dr. R. W. Shufeldt, U. S. Army. Reprint from the Proceedings U. S. National Museum.

Supra—Public Lithotomy. A historical sketch by Charles W. Dulles, M. D. Philadelphia.

Address in Medicine Delivered Before the State Medical Society of Pennsylvania, by Frank Woodbury, M. D. 1887.

On the Physiology of the Heart of the Snake. By T. Wesley Mills, A. M., M., D. Reprint from the Journal of Anatomy and Physiology.

A Physiological Basis for an Improved Cardiac Pathology. By T. W. Mills, M. D. Reprint from the Medical Record.

Monthly Bulletin Iowa State Board of Health. Vol. I., No. 5. Des Moines.

The Sanitary Inspector. A monthly Journal published by the Maine State Board of Health. Augusta.

Public Health in Minnesota. The official publication of the State Board of Health. Red Wing.

University of Toronto. Medical Faculty Calendar Session. 1887-8.

Natural Law in the Business World. By Henry Wood. Lee & Shepard, Boston, 1887.

The Reins and Whip. An Illustrated magazine of general information to all who are interested in the horse and kindred subjects. Published by the Reins and Whip Publishing Co., Phila. Price $2 per year.

